# Screening with magnetic resonance imaging, mammography and ultrasound in women at average and intermediate risk of breast cancer

**DOI:** 10.1186/s13053-017-0064-y

**Published:** 2017-03-01

**Authors:** Tomasz Huzarski, Barbara Górecka-Szyld, Jowita Huzarska, Grażyna Psut-Muszyńska, Grażyna Wilk, Robert Sibilski, Cezary Cybulski, Beata Kozak-Klonowska, Monika Siołek, Ewa Kilar, Dorota Czudowska, Hanna Janiszewska, Dariusz Godlewski, Andrzej Mackiewicz, Joanna Jarkiewicz-Tretyn, Jadwiga Szabo-Moskal, Jacek Gronwald, Jan Lubiński, Steven A. Narod, the Polish Hereditary Breast Cancer Study Group, T Byrski, T Dębniak, A Tołoczko-Grabarek, O Oszurek, M Michalak, H Rogowska-Droś, E Szatkowska, R Kulus, W Kwiecień, L Szyiński, I Winiarska, P Kasprzak, L Tomiak-Rówińska, M Kosterska-Spalska, T Dróżdż, P Sosnowski

**Affiliations:** 1 Department of Genetics and Pathology, International Hereditary Cancer Center, Pomeranian Medical University, Szczecin, Poland; 2 Department of Radiology, Pomeranian Medical University, Szczecin, Poland; 3 Euro-Medic Diagnostics Poland Ltd, Szczecin, Poland; 4 Oncology Diagnostic Center, Zielona Góra, Poland; 5 Regional Oncology Center, Kielce, Poland; 6 Department of Oncology, District Specialist Hospital, Świdnica, Poland; 7 Oncology Diagnostic Center, Legnica, Poland; 8 Department of Clinical Genetics, Collegium Medicum, Nicolaus Copernicus University, Bydgoszcz, Poland; 9 Prophylactic and Epidemiology Cancer Center, Poznań, Poland; 10 Department of Cancer Immunology, Poznan University of Medical Sciences, Greater Poland Cancer Centre, Poznań, Poland; 11 District Specialist Hospital, Toruń, Poland; 12 Department of Radiology, Regional Oncology Hospital, Bydgoszcz, Poland; 13 Women’s College Research Institute, Women’s College Hospital and the University of Toronto, 76 Grenville Street, 6th Floor, Toronto, ON M5S 1B2 Canada

**Keywords:** Breast cancer, *CHEK2*, Magnetic resonance imaging, Mammography, Ultrasound, Screening

## Abstract

**Background:**

The addition of MRI to mammography and ultrasound for breast cancer screening has been shown to improve screening sensitivity for high risk women, but there is little data to date for women at average or intermediate risk.

**Methods:**

Two thousand nine hundred and ninety-five women, aged 40 to 65 years with no previous history of breast cancer were enrolled in a screening program, which consisted of two rounds of MRI, ultrasound and mammography, one year apart. Three hundred and fifty-six women had a *CHEK2* mutation, 370 women had a first-degree relative with breast cancer (and no *CHEK2* mutation) and 2269 women had neither risk factor. Subjects were followed for breast cancer for three years from the second screening examination.

**Results:**

Twenty-seven invasive epithelial cancers, one angiosarcoma and six cases of DCIS were identified over the four-year period. Of the 27 invasive cancers, 20 were screen-detected, 2 were interval cancers, and five cancers were identified in the second or third follow-up year (i.e., after the end of the screening period). For invasive cancer, the sensitivity of MRI was 86%, the sensitivity of ultrasound was 59% and the sensitivity of mammography was 50%. The number of biopsies incurred by MRI (*n* = 156) was greater than the number incurred by mammography (*n* = 35) or ultrasound (*n* = 57). Of the 19 invasive cancers detected by MRI, 17 (89%) were also detected by ultrasound or mammography.

**Conclusions:**

In terms of sensitivity, MRI is slightly better than the combination of mammography and ultrasound for screening of women at average or intermediate risk of breast cancer. However, because of additional costs incurred by MRI screening, and the small gain in sensitivity, MRI screening is probably not warranted outside of high-risk populations.

## Background

Several studies have demonstrated that annual magnetic resonance imaging (MRI) is a more sensitive means of screening for breast cancer than annual mammography for women at high risk of cancer [1–10]. These studies have focused on women at high risk of cancer due to a *BRCA1* or *BRCA2* mutation, or at moderate risk due to a family history. It is not clear if MRI should be used to screen women at average or intermediate risk. It is possible that MRI performs differently in women with and without BRCA mutations because of the differences in cancer risk and also potentially because of the difference in the natural history of cancers in the two groups. Most cancers in non-carriers or in *CHEK2* carriers are ER-positive [11] whereas cancers in *BRCA1* carriers are usually triple-negative [12] and these are relatively aggressive in their natural history [13]. Women with a truncating CHEK2 mutation have a breast cancer risk that is about three fold higher than average and women with both a family history and a CHEK2 mutation have a five-fold increased risk [14]. Missense mutations in CHEK2 confer a 1.4 fold increase in breast cancer risk [14].

There are many relevant issues relating to expanding MRI screening to average - and moderate-risk populations, including the costs of screening, technical considerations including the availability of MRI-guided biopsy and the possibility of over-diagnosis. It is important to establish whether or not the gain in sensitivity obtained with MRI screening in terms of cancers detected over and above mammography (or a combination of mammography and ultrasound) justifies the expense. Ultimately the value of MRI screening should be determined by demonstrating an improvement in breast cancer mortality among women undergoing regular MRI screening.

We conducted a multicenter observational study in Poland wherein we followed 2995 women (2269 at average risk and 726 at moderately elevated risk) who were screened with three screening modalities (MRI, mammography, ultrasound) on two occasions one year apart and then were followed for an additional three years. We estimate the risks of prevalent cancers, incident cancers and interval cancers in the cohort.

## Methods

### Study population

The study group consisted of women at average risk of cancer and women with a moderately increased risk of cancer due to an inherited *CHEK2* mutation or a first-degree relative with breast cancer. To identify *CHEK2*-positive patients, the records of the hereditary cancer clinic of the Pomeranian Medical University were reviewed. Women who were between the ages of 40 and 65 and who had no previous history of breast cancer or of another cancer and were known to carry a *CHEK2* mutation (truncating or missense) were invited to participate. Through this process, 496 *CHEK2* carriers were invited, of whom 356 (72%) agreed to participate. The remaining study participants were recruited through a newspaper campaign conducted in different regions of Poland. Women with and without a family history of breast cancer were eligible to participate. Through this means, the study cohort was expanded to include an additional 2639 women at average risk, including 370 with a family history of breast cancer and 2269 with no family history of breast cancer. The distribution of patients was as follows (Szczecin 758; Olsztyn 604; Zielona Góra 463; Bydgoszcz 100; Toruń 111; Kielce 418; Poznań 142; Świdnica 239; Legnica 160). A blood sample was taken from each woman for DNA extraction. Genetic testing was done for four *CHEK2* mutations (I157T, IVS2 + 1G > A, 1100delC and del5395) and for three *BRCA1* mutations that are founder mutations in Poland (5382insC, C61G and 4153delA) [14]. We included those with a CHEK2 mutations but women with a *BRCA1* mutation were excluded.

### Study protocol

Women were screened twice, one year apart and were followed for three more years for incident breast cancers. Women were included in the study if they had all three examinations (MRI and ultrasound and mammography) at the first round. The ultrasound examination and mammography either took place the day before the MRI examination or on the same day. In some cases the radiologist had access to the MRI results at the time the ultrasound was conducted, but this was uncommon. Clinical breast examination was performed by the radiologist on a routine basis, but was not evaluated formally in this analysis. Ethics approval was obtained by the institutional review board of the Pomeranian Medical University and by all participating institutions. All participants provided written informed consent.

The imaging studies were performed in nine different institutions (see above). MRI screening in each center was performed using General Electric Signa HDXT 1.5 Tesla scanner with gadolinium enhancement. The images generated by the ultrasound and mammography examinations were interpreted locally and the images generated by the MRI were interpreted centrally in Szczecin by two reference radiologists (with the exception of the images generated in Kielce and Bydgoszcz which were interpreted locally). All imaging studies were read by a radiologist affiliated with the study and were classified using the American College of Radiology Breast Imaging Reporting and Data System (BI-RADS) as follows: 0-further information or workup required; 1-negative; 2-benign finding; 3-probable benign finding (short follow-up interval required); 4-suspicious abnormality; 5-highly suggestive of malignancy [14]. If any imaging modality was scored as BI-RADS 5, a biopsy was performed. If the modality was scored as BI-RADS 0 or 4, then a biopsy was not automatically recommended, but was done at the discretion of the treating physician and radiologist, based on the interpretation of all three screening modalities and the physical examination. In some cases, one or more screening test was repeated. In most cases, core biopsies and excisional biopsies were performed under ultrasound or stereotactic guidance. For BI-RADS 5 lesions that were only visualized on MRI-only, an excisional biopsy or quadrantectomy was performed. MRI-guided biopsy was not available. The biopsy specimens were reviewed by pathologists with expertise in breast cancer. Details of cancers detected were collected from hospital medical records and pathology reports. Cancers were divided into ductal carcinoma in situ (DCIS) and invasive cancer. There was one non-epithelial cancer detected (angiosarcoma). Cancers detected on the first screening round were categorized as *prevalent* cancers. Cancers detected on the second screening round were categorized as *incident* cancers. Cancers detected between screening rounds or within one year of the last screen were categorized as *interval* cancers. Cancers detected in the second or third year post-screening were categorized as *post-screening* cancers. These cancers were not considered in the evaluation of screening parameters (e. g. sensitivity) but were included in order to estimate the incidence rate of cancers in the cohort.

### Follow-up

Women were contacted by telephone or by mail annually to inquire if they had been diagnosed with breast cancer in the three-year period following the second screening round. For 80% of the women who entered the study, follow-up was at least two years and for 76% of the women follow-up was three years after the second screening round. No screening MRI was done in the follow-up period. In some cases, the woman may have had screening mammography in the follow-up period outside of the study, but these details were not collected. Further, details on clinical breast examinations were not collected. For those women who developed cancer, details of the cancer were retrieved from medical records.

Women with a BI-RADS 4 abnormality detected on MRI were followed for four to five years from the time of the abnormal MRI (until July 2015) in order to interpret the clinical impact of having a BI-RADS 4 abnormality without a biopsy on subsequent cancer risk. Follow-up was by telephone interview to the patients herself and inquiring about new diagnoses of breast cancer.

### Sensitivity and positive predictive value

We collected information on both invasive cancers and in situ cancers. Sensitivity was calculated for invasive epithelial cancers only. The sensitivity of a given modality was defined as the number of biopsy-proven cancers detected by that modality, divided by the total number of cases detected by all modalities, plus the interval cancers. The positive predictive value was the ratio of the number of biopsy-proven cancers detected by a given modality (BI-RADS 0,4 or 5) divided by the number of biopsies performed that were occasioned by a positive screening test by the same modality. In the cost analysis, the costs used were the actual prices which were negotiated between the Pomeranian Medical University, with the Polish Ministry of Science and Euromedic. The cost analysis is restricted to the costs of screening only (and does not include the costs of biopsies or subsequent clinical care).

## Results

### Patients

A total of 2995 women completed the first round of screening (Table 1). The mean age at entry was 52.5 years (range 40 to 65 years). Of these women, 458 (15%) had a first-degree relative with breast cancer and 356 (12%) had a *CHEK2* mutation (49 truncationg and 307 missense). 51 women had both a first-degree relative with breast cancer and a CHEK2 mutation. Over the four year study period, breast cancer was diagnosed in 34 women, including six cases of ductal carcinoma in situ, one angiosarcoma and 27 invasive breast cancers. The following analyses are limited to the 27 invasive breast cancers.

**Table 1:** Characteristics of 2995 study patients

	Number	Percent
Age at study entry		
40–49	1022	34.1%
50–59	1537	51.3%
60+	436	14.6%
Mean age	52.5	
Family history		
Positive	458	15.3%
Negative	2537	84.7%
*Chek2* mutation		
Truncating	48	1.6%
Missense	308	10.4%
Either	356	12.0%
Neither	2616	88.0%
Missing	23	

In the first round of screening, 13 invasive cancers were detected (prevalence 0.4% or 430 per 100,000) (Table 2). Six of the 13 cancers were palpable, five of 13 were above 2cm in size and three of 13 were node- positive. After one year, the 2982 remaining women were invited for a second screen. Of these, 2565 women (86%) participated in the second round of screening and 417 women (14%) did not return. Of the 2565 women who returned, 2327 women had a second MRI (91%), 2391 had a second ultrasound (93%) and 2329 had a second mammography (91%). One interval cancer was detected between the first and second screening round. This was a 3 cm, node-positive cancer detected in a 50 year old woman. There were seven invasive cancers detected at the second screening round (incidence 300 per 100,000 per year) one of seven was palpable, one of seven was above 2cm in size and two of seven were node-positive.

**Table 2:** Numbers of invasive breast cancers detected by different screening modalities during the screening period

	All	Mammography		Ultrasound		MRI		Any screening	
	Number of cancers	Detected	%	Detected	%	Detected	%	Detected	%
First screen	13	9	69	8	62	13	100	13	100
Interval 1	1	0	-	0		0	-	0	-
Second screen	7	2	29	5	71	6	86	7	100
Interval 2	1	0	0	0		0	-	0	-
Any	22	11	50.01	13	59.1	19	86.4	20	90.9

Of the 2565 women who participated in the second screening round, 2335 women were followed for an additional two years and 2273 women were followed for an additional three years through telephone interview and a mailed questionnaire. In the first year after the screening period (year three) there was only one invasive cancer diagnosed, in a 52 year old woman. It was 3.0 cm in size and node negative. She had been screened with MRI/ultrasound/mammography eight months prior to diagnosis. In the second year post screening, four additional cancers were diagnosed; on average these were 1.7 cm in size and one was node positive. In the third year after screening there was only one breast cancer diagnosed in the left breast of a 53-year old woman. She also had a breast cancer detected in the first round of screening in the right breast. The second (contralateral) cancer was detected by the woman herself and mammography was negative. The cancer was 2.2 cm in size and was node-positive. In total, after the second screen, six cancers detected among 2335 women contributing 6643 person-years of follow-up (incidence 86 per 100,000 per year).

### Screening parameters

#### Sensitivity

Of the 22 invasive cancers detected during the screening period (first screen to one year after second screen) 20 were detected by one of the screening modalities (eight were palpable and 14 were non-palpable) and two were interval cancers. The highest sensitivity was seen with MRI (86%), followed by ultrasound (59%) and mammography (50%). Eighteen of the 22 cancers were detected by either mammography or ultrasound and the sensitivity of mammography and ultrasound in combination (77%) was only slightly inferior to that of MRI (86%). This combination identified one cancer that was missed by the MRI. There were two cancers that were identified by the MRI that were not identified by any other screening modality. These were diagnosed in a 51-year old and a 66-year old woman and were 0.8 cm and 1.1 cm respectively. Both cancers were node-negative, non-palpable, ER-positive and HER2-negative.

#### Positive predictive value

In the entire study, 571 women (19.1% of total) had an abnormal examination by at least one screening modality (BI-RADS 0, 4 or 5). Two hundred and four women (36%) underwent a total of 216 biopsies, including all women with a BI-RADS 5 anomaly, 139 women with a BI-RADS 4 anomaly and 65 women with a BI-RADS 0 anomaly. These 204 women represent 6.8% of the entire study population. On 26 occasions (12.0%) the biopsy revealed cancer (21 invasive and four DCIS, one angiosarcoma) and on 190 occasions (88.0%) the biopsy revealed either normal tissue or benign disease. Overall, the positive predictive value was 12.1% for MRI, was 14.2% for ultrasound and was 31.4% for mammography. There were 216 biopsies performed. There were 156 biopsies performed in women with an abnormal MRI, followed by 57 with an abnormal ultrasound and 35 women with an abnormal mammogram.

### Breast cancers

#### Detection rates

Overall, 22 invasive breast cancers were detected during the screening period (20 screen-detected and two interval cancers). Two of the women had a positive family history of breast cancer and five women had a CHEK2 mutation (one case had both) (Table 3). Assuming a baseline risk of that of women between the age of 40 and 50 without a mutation and without a family history (0.26%), the relative risk associated with a *CHEK2* mutation was 5.4 (95% CI 1.1 to 2.8) and that associated with a family history of breast cancer (first-degree relative) was 1.7 (95%CI 0.2 to 12). The detection rate was 0.43% at the first screening round (prevalent cancers cancers) and was 0.27% at the second screening round (incident cancers).

**Table 3:** Proportions of subjects with invasive breast cancer identified, by risk group

	Number of women screened	Cancers detected	Rate of detection	Sensitivities of different screening modalities			
				Mammography	Ultrasound	M and US	MRI
Family history positive	458	2	0.4%	50%	50%	100%	100%
*CHEK2* mutation	353	5	1.4%	60%	80%	100%	100%
No risk factors	2269	16	0.7%	53%	56%	75%	81%
40–49	1022	5	0.5%	60%	80%	100%	100%
50–59	1537	11	0.7%	64%	45%	71%	82%
60–65	436	6	1.4%	40%	67%	83%	830%
All	2995	22	0.7%	57%	59%	82%	86%

*M* mammography

*US* ultrasound

### Tumor characteristics

The mean size of the 22 invasive cancers was 1.6 cm (range, 0.5 to 3.0 cm). The mean sizes of prevalent and incident invasive tumors were 1.7 cm and 1.2 cm respectively. Three out of 13 prevalent cancers were node-positive and two of seven incident cancers were node-positive (Table 4).

**Table 4:** Characteristics of invasive breast cancers detected during screening period

	All subjects	MRI	Ultrasound	Mammography	Mammography and/or ultrasound
	(*N* = 22)	(*N* =19)	(*N* =13)	(*N* = 12)	(*N* = 18)
Size					
0–0.9 cm	4	3	3	1	3
1.0–1.9cm	10	10	8	5	9
2.0–2.9cm	6	6	2	6	6
3.1 + cm	2	0	0	0	0
Mean	1.6	1.5	1.3	1.8	1.6
Node status					
positive	6	3	5	2	5
negative	16	16	8	10	13
ER					
Positive	18	16	10	10	14
Negative	4	3	3	1	4
PR					
Positive	15	13	7	7	11
Negative	4	3	3	2	4
Not done	3	3	3	3	3
HER2					
Positive	3	2	1	2	2
Negative	19	17	12	10	16

### Long term follow-up of women with abnormal MRI (BI-RADS 4)

In our study, a biopsy was performed on all patients with an MRI BI-RADS 5 lesion, but not on all patients with MRI BI-RADS 4 lesion. This was either for a technical reason or due to patient preference or because of the radiologist’s interpretation of the other two imaging studies done concurrently. Of the 192 women who had a BI-RADS 4 (suspicious) lesion detected on MRI, 87 women had a biopsy and 11 breast cancers were found (12.6%). The 76 women who had a negative biopsy were followed for 4.0 years on average from the date of the abnormal MRI (BI-RADS 4) and two cases of invasive cancer were reported. There were also 105 women who had an abnormal MRI and who did not have a biopsy; these women were followed for an average of 4.3 years and two women were diagnosed with breast cancer. One was diagnosed with an invasive 4 cm node-positive cancer 55 months from the abnormal MRI. The other was diagnosed with a breast cancer in the breast contralateral the MRI abnormality, eight months after the initial abnormal MRI. The tumor was 5.0 cm and the patients was node-positive. In summary, of 192 women in the study who had a BI-RADS 4 abnormality, 11 breast cancers were detected at the time of screening and three cancers were diagnosed in the four year period following the abnormal MRI (one of these was diagnosed within the four year study period). Of the 151 women with an MRI BIRADS 4 abnormality and no other screening abnormality, four breast cancers were found, two at the time of the MRI and two thereafter. Of these, two were less than two centimeters and three were node negative.

## Conclusion

In this study of 2995 women at average or intermediate risk of breast cancer, we found the combination of mammography and ultrasound to be nearly equivalent to annual MRI. Of the 22 women who were diagnosed with invasive breast cancer over the two-year screening period, 19 were visualized with MRI and 17 were visualized with ultrasound and/or mammography. We report a high overall sensitivity (91%) and a very low proportion of interval cancers (9%) – both are indicators of an effective screening program. The average size of the breast cancers was small (1.6 cm) and the incident cancers were smaller, on average (1.2 cm) than were the prevalent cancers (1.7 cm). Fourteen of the 22 invasive cancers were detected in the cohort at a non-palpable stage and no cancer was detected solely by physical examination. This was an observational study and the management of the patients found to have an abnormal lesion was at the discretion of the treating physicians.

We also identified six cases of DCIS. There is no consensus regarding the value of identifying Stage 0 breast cancers in the context of screening and in order to guard against over-interpretation, we have separated these from the invasive cancers.

In this study, there is little evidence to support the position that annual MRI screening should be recommended for women from the average/moderate risk population who are being screened with annual mammography. Two cancers were identified through MRI screening that were missed by the other screening modalities. To some extent, the small number of cancers detected through MRI alone (*n* = 2) might be a reflection of the reluctance of the radiologist or surgeon to biopsy a patient with an abnormal lesion detected on MRI (BI-RADS 4) when there was no corresponding lesion detected on mammography or ultrasound. However, among the 105 women with a BI-RADS 4 abnormality who did not have a biopsy, only one cancer was detected clinically in the three year period following the positive MRI test and this was detected in the contralateral breast. The other cancer was diagnosed 55 months post-MRI and the length of time elapsed suggests that this was an unrelated (new) event. These observations suggest that the reluctance to biopsy these women initially did not result in the failure to diagnosis many cancers that would have been clinically apparent in the three year interval following the second MRI examination. The lack of incident cancers in this group of 105 women in the three years following the abnormal MRI abnormal result suggests that there were some false positive MRI tests or that some cancers may be have been due to over-diagnosis. Over-diagnosis is defined as a cancer that presents on screening but would not otherwise become apparent in a patient’s lifetime. Miller et al estimate in the Canadian NBSS study [15] that approximately one-half of non-palpable breast cancers detected solely by mammography are examples of over-diagnosis and there is no reason to suppose the proportion to be less among MRI-detected cancers (which on average are smaller) should be less than this.

It is important that the results of screening studies be interpreted in the context of the level of risk of the participating subjects. Studies of women at high risk (i.e., *BRCA* mutation carriers) in general are more supportive of intensive screening than are studies of women at average or intermediate risk. In *BRCA1* and *BRCA2* carriers, the cancer prevalence (and incidence) is much higher than in the cohort studied here (approximately 6-fold higher) and the use of MRI as a screening tool can be justified. For example, in the recent paper by the Toronto group of 496 *BRCA1* and *BRCA2* carriers [16], 57 cancers were identified in 1847 screening rounds (31 per 1000) compared to 26 cancers in 5319 screening rounds (5 per 1000) in the present study. The sensitivity of MRI was 86%, versus 19% for mammography alone. In a Dutch trial of MRI screening for high-risk women [10], conducted from 1999 to 2006, 47 cancers were found among 594 *BRCA* mutation carriers, compared to 34 cancers among 1558 women at familial risk, but without a mutation. The detection rates for invasive cancers were 26.5 and 4.8 cancers per 1,000 woman-years in carriers and non-carriers respectively (in our study the equivalent rate was 3.5 invasive cancers for 1,000 woman-years). They reported sensitivities of 71% and 41% for MRI and mammography respectively. We excluded women with a Polish founder mutation in *BRCA1* from this cohort; three founder mutations comprise the majority of BRCA mutations in Poland. It is possible that there are other mutation carriers in this cohort but given the rarity of non-founder mutations in the Polish population and the fact that study subjects were unaffected, we expect the number of these to be very low.

In the present study 25 cancers (DCIS and invasive) were found through 216 biopsies (positive fraction 11.6%). In an early study of women with a BRCA1 or BRCA2 mutation, 33 biopsies revealed seven cancers (21.2%) [17]. This difference is expected; given the lower prevalence of cancer in the average risk women compared to high risk women, the positive predicive value will be lower in the former group.

In a recent report from the UK, (the FH01 study), the sensitivity of mammography alone was reported to be 77% for screening of 6710 young women at intermediate risk [17]. They estimated a mortality reduction of approximately 20% associated with mammography. In that study, 21% of cancers were interval cancers (compared to 5% in the present study), 32% of cancers were node-positive (compared to 24% in the present study) and 30% were above 2 cm (compared to 33% in the present study). To some extent, these differences may be due to different age distributions. All patients in the FH01 study were under 50, compared to 34% of the subjects in our study.

In the ACRIN study, 2662 women at high risk underwent 7443 mammogram and ultrasound screening tests over two years (three annual screens). Thirty three cancers were detected by mammography alone, 32 were detected by ultrasound alone, 26 were detected by both modalities and nine were not detected. The overall detection rate was 17 per 1000 woman-years. At the end of screening 612 women had an MRI and nine more cancers were detected (1.5%). The authors concluded that the routine use of MRI was not justified in this intermediate risk population.

The data presented here do not support adding MRI screening to routine mammography and ultrasound for women at average risk at this time. The addition of MRI to the combination of ultrasound and mammography in this cohort of 2995 women led to the identification of two additional cancers. The resources expended to identify these two cancers were 5319 MRI examinations and 64 biopsies. This is equivalent to 3.7 cancers detected per 10,000 MRI examinations. Assuming a cost of 155 dollars per MRI (the actual reimbursed cost), this is equivalent to 400,000 dollars per additional cancer detected in Poland, but is expected to be much higher than this in North America or western Europe. In this study, the cost of the ultrasound and mammography were 40 dollars per examination. Based on these prices, the total costs of screening 10,000 women would be 400,000 dollars for a combination of mammography and ultrasound and 1,550,000 using MRI alone. Our study is based on a relatively small number of detected cancers, and we recommend that further research be done in this area. It is important that the value of screening MRI ultimately be judged on studies with cancer mortality as the endpoint, but to date, there are no published studies with mortaity as the endpoint. It is also important to consider that there are potential harms associated with screening, particularly in low and average risk populations, such as increased numbers of confirmatory tests, anxiety, surgical morbidity and (in the case of over-diagnosis) unnecessary sugeries. Given the very low risk of cancer we observed in the follow-up period of women with BIRADS 4 lesion who did not undergo a biopsy, futher studies which consider the potential harms of overdiagnosis associated with MRI screening are warranted. Given the high cost of MRI examinations, until supporting evidence is available, we concur with other investigators [17] that the addition of MRI to mammography and ultrasound for screening women at intermediate risk of breast cancer is not appropriate.

## Abbreviations

BI-RADS: Breast imaging reporting and data system
DCIS: Ductal carcinoma in situ
MRI: Magnetic resonance imaging
